# Genome-wide polysomal analysis of a yeast strain with mutated ribosomal protein S9

**DOI:** 10.1186/1471-2164-8-285

**Published:** 2007-08-21

**Authors:** Lilach Pnueli, Yoav Arava

**Affiliations:** 1 Faculty of Biology, Technion – Israel Institute of Technology, Haifa 32000, Israel

## Abstract

**Background:**

The yeast ribosomal protein S9 (S9) is located at the entrance tunnel of the mRNA into the ribosome. It is known to play a role in accurate decoding and its bacterial homolog (S4) has recently been shown to be involved in opening RNA duplexes. Here we examined the effects of changing the C terminus of S9, which is rich in acidic amino acids and extends out of the ribosome surface.

**Results:**

We performed a genome-wide analysis to reveal effects at the transcription and translation levels of all yeast genes. While negligible relative changes were observed in steady-state mRNA levels, a significant number of mRNAs appeared to have altered ribosomal density. Notably, 40% of the genes having reliable signals changed their ribosomal association by more than one ribosome. Yet, no general correlations with physical or functional features of the mRNA were observed. Ribosome Density Mapping (RDM) along four of the mRNAs with increased association revealed an increase in ribosomal density towards the end of the coding region for at least two of them. Read-through analysis did not reveal any increase in read-through of a premature stop codon by the mutant strain.

**Conclusion:**

The ribosomal protein rpS9 appears to be involved in the translation of many mRNAs, since altering its C terminus led to a significant change in ribosomal association of many mRNAs. We did not find strong correlations between these changes and several physical features of the mRNA, yet future studies with advanced tools may allow such correlations to be determined. Importantly, our results indicate an accumulation of ribosomes towards the end of the coding regions of some mRNAs. This suggests an involvement of S9 in ribosomal dissociation during translation termination.

## Background

The ribosome is a large RNA-protein complex having multiple roles in the translation process. The RNA moiety in eukaryotes includes four RNA molecules that provide the structural scaffold for the functional sites of the ribosome, and interacts with various proteins of the translation machinery. The protein moiety is composed of more than 70 proteins, located mostly at the ribosome's periphery. These proteins were shown to play various roles, including assistance in ribosome assembly, interaction with translation initiation factors, stabilization of the ribosome structure, and more [1,2].

The yeast ribosomal protein S9 (*sup46*) is known to play a role in accurate decoding [3,4]. Mutagenesis analysis yielded a general (omnipotent) suppressor phenotype, characterized by suppression of termination by all three stop codons, as well as misreading of various codons [5-8]. Some of these mutations are characterized by an increase in the isoelectric point of S9, suggesting a defect in acidic amino acids [9]. Recent structural studies in prokaryotes and eukaryotes revealed that S9 is located at the head of the small ribosomal subunit near the entrance tunnel of the mRNA leading into the decoding center [10-12]. Part of the protein is in close contact with the rRNA, while other regions extend out of the ribosome. The position of the bacterial homolog of S9 (S4) prompted Yusofova et al. to suggest a role in opening the secondary structure of the incoming mRNA [11]. Indeed, it has been recently shown that prokaryotic ribosomes having mutations in S4 (the *E. coli *homolog of S9) possess a lower ability to unwind RNA duplexes [13]. Thus, S9 might exhibit a helicase activity, which may be related to its role in accurate decoding.

**Figure 1:**
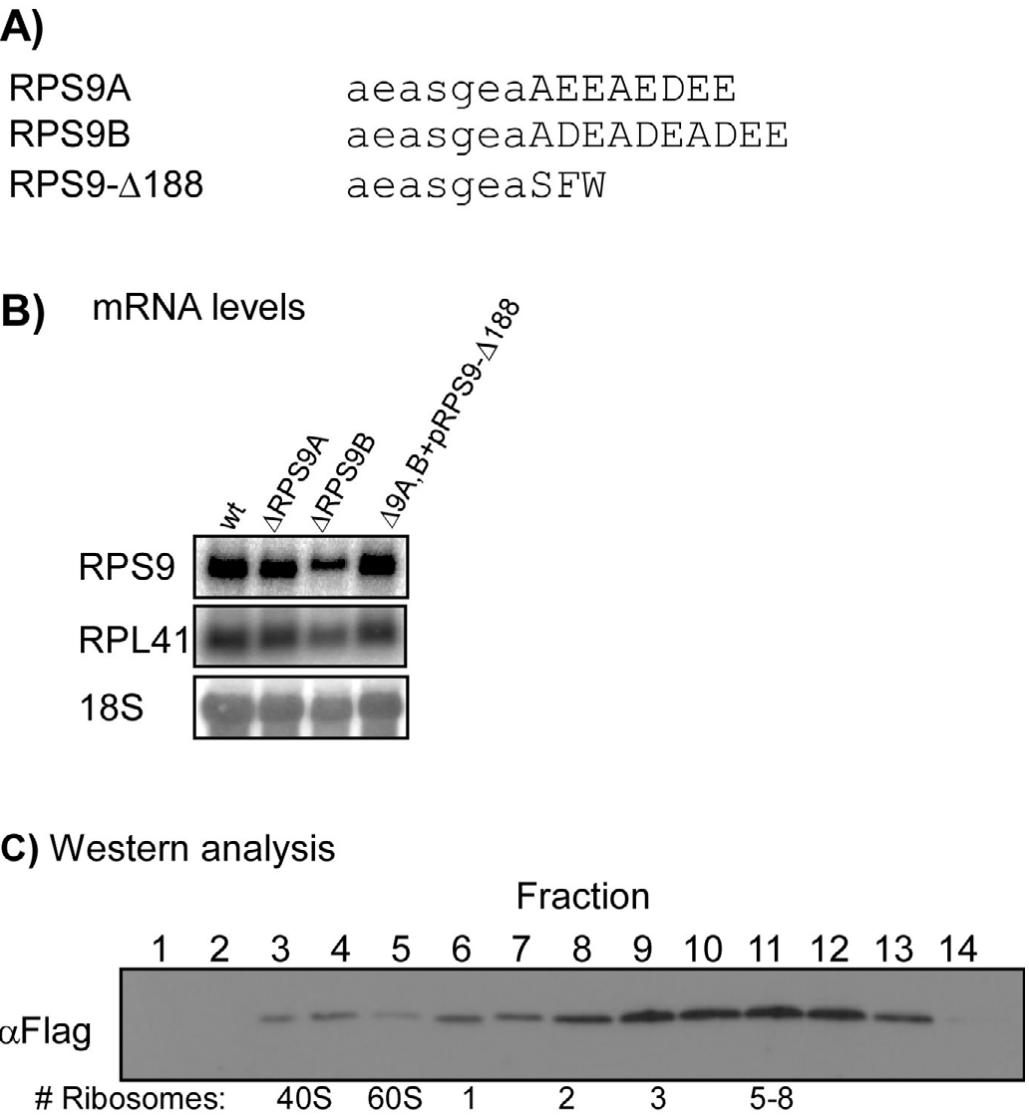
**Expression of *rps9-Δ188***. A) Sequence of the C terminus of S9A, S9B and the deleted protein rps9-Δ188. Lowercase letters indicate identical residues. B) Northern blot analysis of RNA samples prepared from the parental strain (wt), strain deleted of only *RPS9A *gene (*ΔRPS9A*), strain deleted of only the *RPS9B *gene (*ΔRPS9B*) and strain deleted of both genes and supplemented with a single-copy plasmid containing *rps9-Δ188*. The membrane was hybridized with probes recognizing *RPS9 *ORF (upper panel), *RPL41 *(middle panel) and 18S rRNA (lower panel). C) Western analysis of the sedimentation profile of *rps9-Δ188*. Cells expressing flag-tagged *rps9-Δ188 *were harvested and the cell extract was separated by velocity sedimentation on a sucrose gradient. The gradient was fractionated into 14 fractions and the proteins of each fraction were subjected to western analysis using anti-flag antibodies. The sedimentation positions of ribosomal complexes were derived from the OD254 profile of the gradient (not shown) and are indicated at the bottom of the panel.

In *S. Cerevisiae*, there are two genes for S9 (*RPS9A *and *RPS9B*) that differ significantly in their transcription levels. While *RPS9B *is highly transcribed, much lower levels of *RPS9A *were detected ([4,14] and Fig. 1B). The encoded proteins, however, are almost identical and differ only in five amino acids, four of which are at the highly acidic C terminus. Deletion of either gene is not lethal, yet the omnipotent suppression phenotype was only shown for mutations in *RPS9B*, and expression of *RPS9A *did not complement the phenotype [4,14]. This suggests that the suppressor phenotype of *RPS9B *is either due to the expression difference or to the slight sequence difference.

Here we characterized the effects of changing the acidic C terminus of S9. We performed a genome-wide polysomal analysis of a strain expressing S9 mutated in its last 10 amino acids. Of the ~800 genes for which we were able to compare the ribosomal association between wild-type and the mutant strain, 40% had a significant change in ribosomal association. Analysis of several physical and functional features of the affected transcripts did not reveal any feature that correlates with the extent of change. Ribosome density mapping (RDM) analysis for four mRNAs with increased association suggests an accumulation of ribosomes towards the 3' end of the mRNA for at least two of them. These results suggest that S9 is involved in ribosomal dissociation at the termination site.

## Results

### Mutation of S9

S9 is an essential protein located at the entrance tunnel of the mRNA into the ribosome. It was implicated in functions related to translation elongation such as decoding accuracy and mRNA unwinding. To further explore functions of S9, we performed partial deletions at various regions of interest. Here we present data regarding a mutation of the last 10 amino acids (Fig. 1). These amino acids include multiple aspartic and glutamic acids (D and E) and form a DEAD-like sequence (yet no other motif of classical DEAD helicases appears in S9). They are located outside the region of interaction with the rRNA (the S4 domain) and extend out of the ribosome surface [4,11,12].

There are two copies of the S9 gene in yeast that express almost identical proteins. The differences between the encoded proteins are only arginine to lysine substitution in position 40, and four amino acids difference in the C terminus. Both genes were deleted by the mating and sporulation of haploids carrying either *RPS9A *deletion or *RPS9B *deletion. A single-copy plasmid carrying the mutated *RPS9A *coding region with 500 bp upstream and 300 bp downstream was inserted into these cells prior to sporulation, and was selected during the sporulation with the appropriate medium. The last 10 amino acids (188–197) were deleted from the mutated S9 and the deletion procedure led to the addition of three amino acids (Ser, Phe and Trp). Although these added amino acids are uncharged, we cannot exclude the possibility that the observed effects are due to their addition. The deletion resulted in the removal of four amino acids differing between S9A and S9B, and the only remaining difference between S9A and S9B is the substitution of basic amino acid at position 40. We therefore refer to the resulting altered protein as S9 rather than S9A and hereinafter designate it as *rps9-Δ188*.

**Figure 2:**
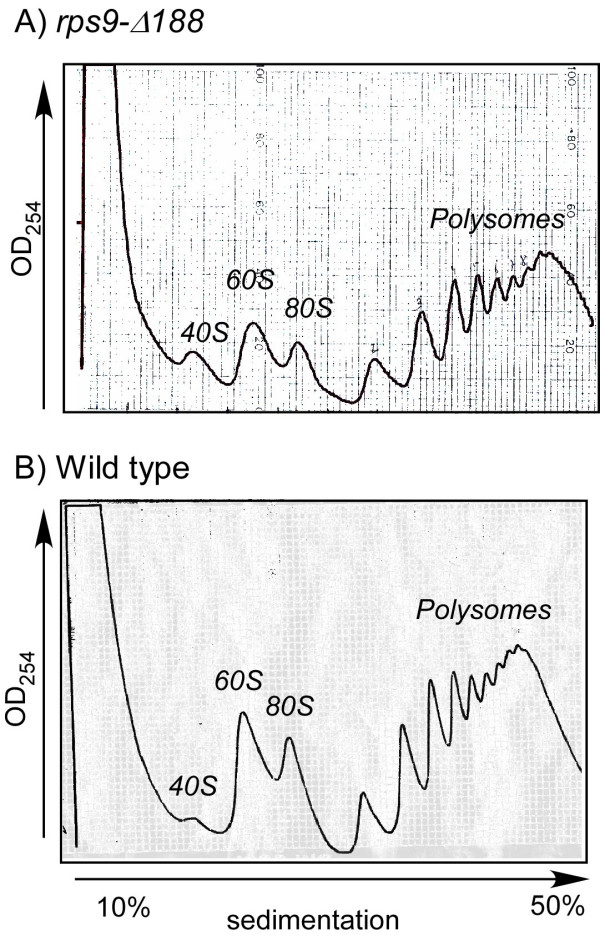
**Polysomal profiles of wild-type cells and cells expressing *rps9-Δ188***. Cells expressing only *rps9-Δ188 *gene (A) or both *RPS9 *genes (B) were grown to the mid-logarithmic phase and harvested. Cell lysates were separated on a 10%–50% sucrose gradient and the OD254 along the gradient was monitored. The sedimentation position of ribosomal complexes (40S, 60S, 80S and polysomes) is indicated on each panel.

The resulting strain exhibited normal growth rates in rich medium (YPD), and northern analysis of *rps9-Δ188 *mRNA levels revealed similar levels to the wild-type strain (Fig. 1B). No accumulation of rRNA intermediates was observed when samples were run on agarose gels, and the ribosomal profile appeared similar to wild-type cells (Fig. 2). Thus, although S9, as several other ribosomal proteins, was shown to be involved in rRNA processing [15], this mutation probably does not interfere with this process. We also generated an isogenic strain in which the mutated S9 was tagged at the N-terminus with a Flag epitope. Western analysis of polysomal fractions collected from this strain revealed its association with fractions corresponding to the small ribosomal subunit, 80S and polysomes (Fig. 1C). This result and the normal growth of the mutant strain indicate that the mutated S9 is inserted into the ribosome. To avoid effects that are due to the highly acidic Flag tag, all subsequent experiments were performed with the untagged *rps9-Δ188*.

### Genome-wide analysis of *rps9-Δ188*

To determine the effects of mutating S9 on a broad spectrum of mRNA sequences, we utilized DNA microarrays carrying ~6,000 known and predicted yeast ORFs. We first describe the results of a transcriptome analysis that characterizes the relative effects on the steady-state mRNAs levels, and then the results of analysis of multiple polysomal fractions aimed at characterizing the effects on ribosomal association.

For transcriptome analysis, RNA was collected from wild-type and *rps9-Δ188 *strains grown on either rich media (YPD) or minimal media (SD plus Met, Lys, Ade). Each of the RNA samples was labeled with red fluorescent dye (Cy5) and hybridized to a DNA microarray together with a reference RNA sample labeled with a green fluorescent dye (Cy3). About 5,000 genes passed the quality selection procedure (see Methods section) in each of the microarrays (Additional File 1). To determine the genes that were affected by the S9 mutation, we compared the changes in expression of the mutant relative to the wild-type strain in each growth condition. The relative expression of only 52 and 97 genes were changed by more than two-fold in YPD and SD media, respectively (Additional File 1). Many of these genes are Ty elements, a repetitive sequence in the yeast genome that experimentally might represent only a single sequence. The small number of genes whose relative abundance had changed indicates that the mutation in S9 has minor effects on the transcriptome profile of the parental strain. In addition, although S9 was shown to be part of the yeast penta-snRNP complex [16], we did not observed any significant change in the relative abundance of intron-containing mRNAs. It should be noted that because of the experimental set-up, these changes are only relative and do not report on global changes (e.g., a strong and similar decrease in abundance for all mRNA).

The observation that more genes are affected in SD medium might be related to the higher transcriptional activity of some metabolic pathways under these conditions. Interestingly, the group of reduced genes in the minimal medium included genes involved in nucleic acid metabolism (mainly adenine synthesis), although the medium included adenine, and none of the adenine pathway genes is mutated in this strain. Consistent with this, we observed that the mutant strain exhibits slow growth phenotype in the absence of adenine in the growth media (Additional File 1). This may suggest an indirect role for S9 in the transcription of genes involved in nucleic acid metabolism, probably by affecting the translation of a transcription factor. Indeed, polysomal profile analysis of the Bas1 transcription factor, which is known to regulate the expression of genes encoding enzymes involved in purine, pyrimidine and histidine biosynthetic pathways [17,18], revealed a decrease in its polysomal association from >80% in the wild-type strain to 57.3% +/- 3.2% in the mutant (data not shown).

To gain insight into the role of S9 during translation, we performed genome-wide analysis of mRNAs separated into multiple polysomal fractions. The *rps9-Δ188 *strain was grown in optimal conditions (YPD), cells were harvested, and large complexes were separated by velocity sedimentation in sucrose gradient. Figure 2A presents the OD 254 profile of the entire gradient that is indicative of the amounts of polysomal complexes. As can be observed, there is a strong signal in the region of the gradient that includes polysomal complexes, and no significant differences are observed when comparing the OD profile to the profile of a wild-type strain (Fig. 2B). This indicates that the majority of mRNAs in the cells is associated with ribosomes, and is presumably translated.

To characterize changes in translation of particular mRNAs, we collected multiple fractions along the gradient and analyzed the mRNA content in each fraction by DNA microarray. The first fractions were pooled together and included all complexes up to the size of 80S. This fraction was designated "free mRNA" sample, since it contains molecules that are free of fully assembled ribosome. The remaining part of the gradient, which included mRNAs associated with one or more ribosomes, was fractionated to multiple fractions of equal volume. Five *in vitro *transcribed bacterial RNAs were spiked into each of the fractions at equivalent amounts and were used to normalize for differences in all subsequent steps of the procedure, including RNA purification, labeling with fluorescent dyes, hybridization to DNA microarrays and scanning [19]. This external normalization procedure retains the inherent differences in mRNA amounts at the different fractions, and therefore enables the construction of a sedimentation profile for each gene, which is refractory to global changes in mRNA levels [20]. RNA from each fraction was labeled with red fluorescent dye and hybridized together with an unrelated RNA sample, labeled with green fluorescence as a reference to a DNA microarray. This experimental procedure was repeated three times, and the ratios obtained for all genes are presented in Additional File 2. There are many missing signals for many genes at various fractions. These missing values represent spots that did not pass the quality selection criteria for various reasons, either technical (e.g., bad spotting of the DNA on the microarray) or biological (e.g., no transcripts of that gene in this fraction). For further analysis we selected only those genes that, in general, had only one missing value in their polysomal region of the gradient (see Methods section for details). This selection resulted in ~4,000 genes for which we could assign a "peak fraction", i.e., the fraction having the highest red-to-green signal (Additional File 3). From this group of genes, we filtered in only genes in which the peak fraction repeated itself in two or more experiments (i.e., one or less fraction difference). About 2,800 genes passed this selection criterion, and from the peak fraction and the OD260 of the gradient (Fig. 2) we assigned for each mRNA the average number of ribosomes that it is mostly associated with (Additional File 4).

**Figure 3:**
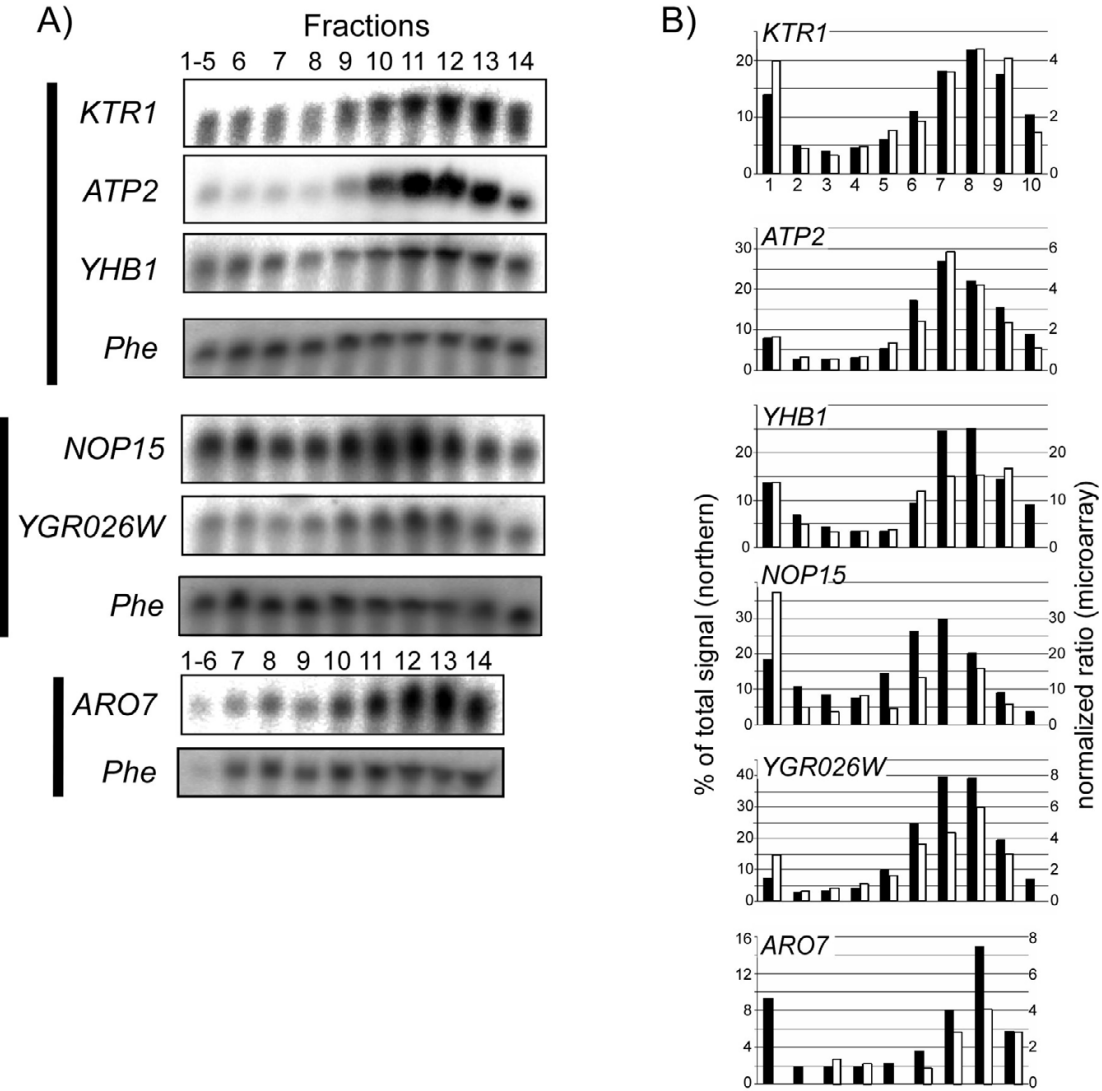
**Comparison of northern blot results with microarrays data**. A) RNA samples from each of the fractions collected for microarray analysis were separated on formaldehyde-agarose gel and subjected to northern blotting. Three blots were prepared, one from each experimental repeat (indicated by bars at the left). Blots were hybridized with probes complementary to the genes indicated at left of each panel and with a probe for the spiked-in Phe RNA. B) Comparison of quantitation results of the northern analysis with microarray data. Black bars represent the northern analysis signal from each fraction normalized to its corresponding signal of the Phe RNA and calculated as a percent of total signal of that mRNA in the gradient. Open bars represent the ratio obtained in the microarray analysis (note that the histogram has two Y-axes) normalized by a signal from the *in vitro *transcribed mRNAs. Fractions where open bars are missing indicate spots that did not pass the quality criteria in the microarray assay. Note that the Y-axis scale of the microarray results differs from gene to gene. This is probably due to differences in their mRNAs abundances compared to the reference sample.

To verify the microarray results using an alternative method, we performed a northern analysis on polysomal fractions of six different mRNAs (Fig. 3). For each gene, the signal obtained from the northern hybridization was normalized to the signal obtained for one of the spiked *in vitro *mRNA (Phe), similar to the normalization procedure performed in the microarray analysis. As can be seen in Figure 3B, the profile obtained by northern analysis in all cases agreed well with the one obtained by the microarray analysis.

To determine whether the mutation in S9 led to changes in ribosomal density of specific mRNAs, we compared the findings to published values of an experiment that was done with an isogenic wild-type strain grown under the same medium and subjected to the same extraction procedure[19]. Overall, the wild-type strain and the mutant strain yielded a similar polysomal profile (Fig. 2 and Fig. 1A in [19]), consistent with no major differences in the experimental procedure. About 800 genes had a reliable ribosomal association value in both the wild-type and the mutant strain (Additional File 5). Not surprisingly, this group includes genes whose transcripts are relatively highly abundant compared to the entire genome of yeast. The median abundance value for this group is 2.2 mRNA copies per cell, while for the entire yeast genome the value is only 1.2 copies per cell. The bias towards abundant mRNA is probably due to the stringent selection procedure, which filters out genes with weak signals or with missing signals from multiple microarrays. This bias probably led to other indirect effects, including the following: 1. An enrichment for genes containing introns (82 of the genes had introns, while in the entire yeast genome the abundance of such genes is <5%). Intron-containing genes have relatively high abundance of mRNAs in yeast. 2. Exclusion of relatively long transcripts (>4,000 nts), as almost all of them are expressed at low levels in yeast. The results and conclusions presented below were also obtained when much lower filtration criteria were employed (data not shown).

The selected genes were divided into seven categories according to the extent of change in their ribosomal association (Table 3 and Additional File 5). For 60% of the genes (486), the S9 mutation had a very small effect of one or less ribosome change. Yet, 40% of the genes had a more pronounced change due to the mutation. To explore the basis for the change in ribosomal association, the genes in each category were compared for various physical and functional properties. For each group, we calculated the average codon bias (either according to codon adaptation index (CAI [21]) or codon bias (CB [22])), GC content, predicted thermal stability (derived using the mfold program [23]) of the 100 nts upstream to the start codon (ΔG of the 5' UTR), predicted thermal stability of the ORF (ΔG ORF), ORF length and mRNA length [24]. We also calculated each group's average abundance and half-life as previously assayed [25] (Table 3). A simulation procedure was performed to determine if there is enrichment for certain parameters in each group. The simulation was based on selecting a similar number of genes at random and calculating the average value for this random set. This was repeated for 10,000 iterations and the distribution of average values was determined. While no general correlations were observed, some parameters appeared to be significantly enriched in two groups: 1) the group of genes with the strongest increase in ribosome density appeared to have a high enrichment for parameters related to expression level – these parameters include mRNA abundance and codon usage (either CAI or CB), which are relatively low compared to the other groups. 2) The genes with the strongest decrease in density appeared to be enriched in parameters related to transcript length, including ORF length and ΔG. Note that ORF length and ΔG strongly correlate with each other (Pearson correlation of 0.98), therefore it is expected that both will perform in the same way. We attempted to normalize for the two parameters (e.g., divide the ΔG by the length of ORF) in order to distinguish between the effects of the two parameters, yet we did not obtain statistically significant results, probably because of the strong linkage between them.

**Table 3:** Various features of groups of affected mRNAs

Ribosome Difference^a^	Number of Genes	CAI	ΔG 5UTR (kcal/mol)	GC content (%)	ΔG ORF (kcal/mol)	ORF length (nts)	WT abundance (copies/cell)	Mutant abundance^c ^(copies/cell)	t_1/2 _(min)
(-4) ≥	24	0.15^b^	14.8	39	285	1189	1.7^b^	1.5	16
(-2)–(-3)	192	0.26	12.8	41	268	1021	4.5	3.6	17.7
(-1)	120	0.25	13.5	42	219	851	5	4.4	16.9
0	307	0.3	12.7	41	327	1354	5.9	4.9	16.5
1	59	0.29	13.2	41	193	766	4.9	3.8	17.1
2–3	87	0.28	14.2	41	198	800	5	4.7	16.2
4 ≤	26	0.24	13.4	40	565^b^	2077^b^	2.8	2.6	18.9
All 815 genes		0.28	13.1	41	281	1075	5.1	4.2	16.9

a – Number of ribosomes in the parental strain (*19*) minus number in *rps9-Δ188 *strain (this study).

b – Values that differ significantly from the rest of the genes.

c – The abundance in the mutant was calculated from the transcriptome analysis by multiplying the relative expression in the mutant by values obtained from the wild-type strain (*25*).

We also utilized the GO mapper tool from SGD in order to determine if any enrichment for specific functional groups exists in any of the affected groups. For this analysis we combined several groups together (i.e. change of two and more ribosomes to either direction) in order to have groups large enough. The lists of genes that changed by two or more ribosomes was subjected to this analysis (Additional Files 6 and 7). We did not observe any specific enrichment in the process, the function or the component that these genes are assigned to.

### S9 mutation leads to increased density at the 3' end

**Figure 4:**
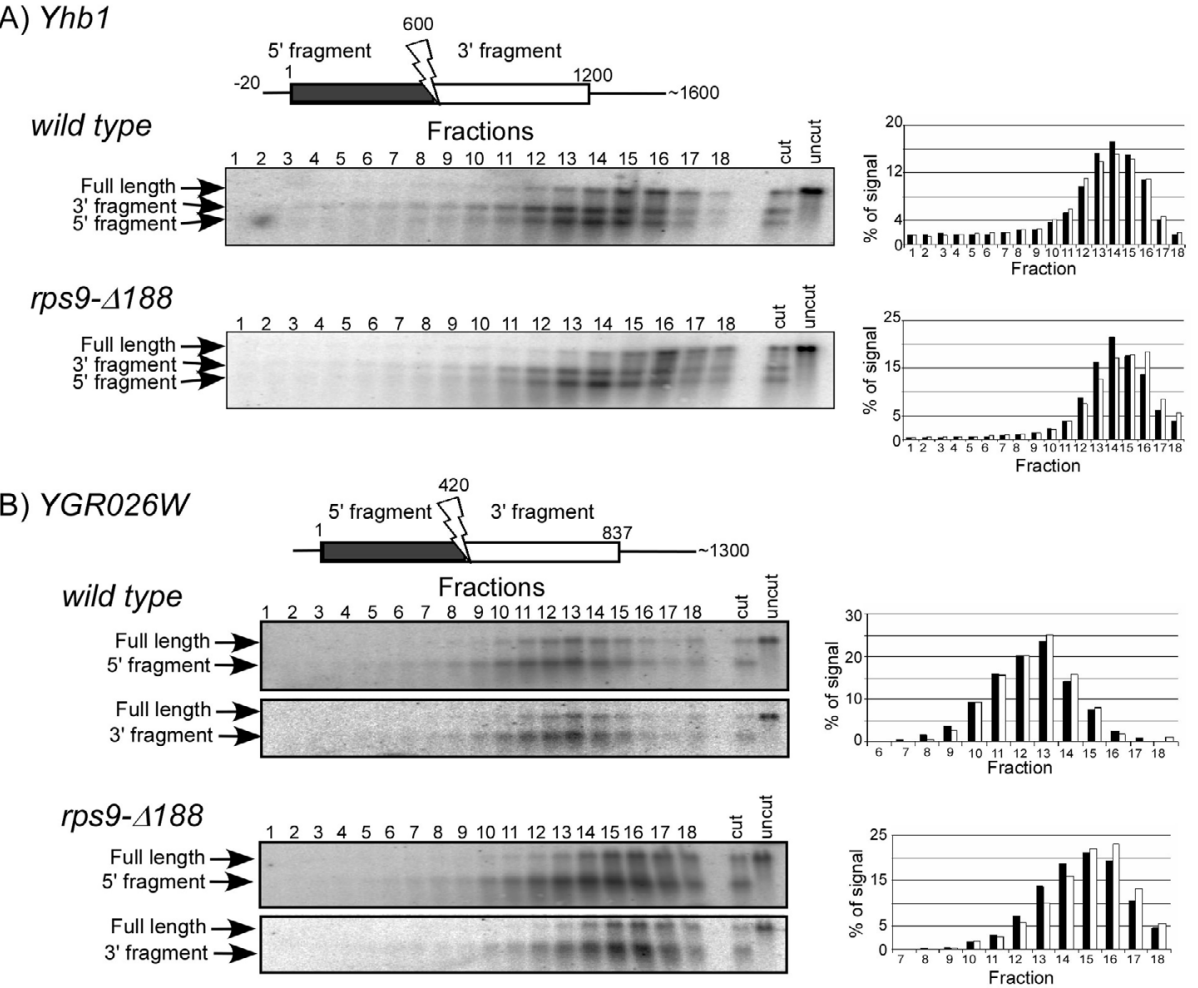
**Comparison of ribosomal association between two halves of an mRNA by RDM**. Polysomal fraction containing the majority of Yhb1 mRNA (A) or YGR026W mRNA (B) was collected from wild-type or *rps9-Δ188 *strains and mixed with RNase H and ODN complementary to the region indicated by an arrow on the schematic presentation of each mRNA. This should lead to a cleavage of the mRNA at the region complementary to the ODN and to result in two fragments: 5' fragment (depicted in black) and 3' fragment (depicted in white). Following the RNase H cleavage reaction, samples were separated on a sucrose gradient into 18 fractions and subjected to northern analysis. Hybridization for Yhb1 (A) was performed using a probe that recognizes the entire open reading frame, therefore both fragments appear in the same panel. Hybridization for YGR026W (B) was performed using probes specific either to the 5' or 3' fragments of the mRNA (upper and lower panels, respectively). Arrows to the left of each panel indicate the migration position of the cleavage product, as well as residual uncut mRNA ("full-length"). Histograms represent the quantitation results of the 5' fragment (black bars) and 3' fragment (white bars) of each mRNA.

To gain insight into the basis of increased ribosomal density, we examined the ribosomal distribution along some of the mRNAs having higher ribosomal density. Increased density at particular regions of the mRNA (e.g., stop codon) will suggest the involvement of S9 at a specific translation step (e.g., termination). We performed a ribosomal density mapping (RDM) analysis on four mRNAs (*Yhb1*, *Aro7*, *YGR026W *and *Nop15*) that appeared to have three or more ribosomes compared to the wild-type strain. For each gene, the polysomal fraction containing most of its mRNAs was isolated from the wild-type and mutant strains, and cleaved at the middle of the coding region by the addition of specific antisense oligodeoxynucleotide (ODN) and RNase H. The cleavage products were separated on a sucrose gradient, and the sedimentation position of each half of the mRNA was determined by northern analysis (Fig. 4). During the cleavage reaction, the mRNA is associated with ribosomes, and therefore the cleavage site is not always accessible to the ODN. Thus, in every experiment there is a fraction of mRNAs that was not cleaved (marked as Full length in all panels) and sediment in the heavy region of the gradient. mRNA molecules for which the cleavage was successful yield two products (5' fragment or 3' fragment), which usually sediment in lighter fractions of the gradient than the full-length as they contain less ribosomes. In the wild-type strain, both halves of each *Yhb1 *(Fig. 4A) and *YGR026W *(Fig. 4B) appear to sediment exactly the same, and the same fraction contains the highest signal for both halves (fraction 14 for *Yhb1 *and fraction 13 for *YGR026W*) (see Table 4 for a summary of the three experiments). This result is consistent with similar ribosomal association on both halves of each message. In the *rps9-Δ188 *strain, however, the peak fraction for the 3' half is in the heavier fractions in both cases: in fraction 16 for *Yhb1 *and fraction 16 for *YGR026W*. This indicates that the 3' half for both genes sediment more heavily than the 5' half, consistent with more ribosomes being present in this region. This result was repeated in three independent experiments, and the sedimentation of the 3' half always appeared heavier than the 5' half. Calculating the weighted-average fraction of the signal indicated an increase in the sedimentation of the 3' fragment in all cases (Table 4). It is difficult to determine the extent of this change in terms of number of ribosomes due to the low resolution in this region of the gradient and the lack of sedimentation markers. In addition, averaging the extent of changes in the different experiments is also not informative since a shift of a single fraction in the heavy region of the gradient is not comparable to a similar shift in the lower region of the gradient. Yet, for all genes and in all repeats, the fragment containing the stop codon (i.e., 3' fragment) sediments as associated with more ribosomes than the upstream fragment (i.e., 5' fragment). It should be noted that although the changes observed by RDM are relatively small, they are significant in this experimental set-up because both halves are the products of the same cleavage reaction, are separated on the same gradient and are analyzed on the same northern blot. Thus, the two halves can be compared directly to each other, and any difference in sedimentation can be directly related to the mRNA fragment and not the experimental set-up.

**Table 4:** Summary of the three experimental repeats of the RDM analysis

		First Repeat^a^		Second Repeat		Third Repeat	
**Gene **(*cleavage site*)	**Resulting fragment**	**Wild-type**	***rps9-Δ188***	**Wild-type**	***rps9-Δ188***	**Wild-type**	***rps9-Δ188***

**YHB1 **(*cleavage at the middle of the ORF*)^b^	5' half	13.7	14.3	13.5	15.2	14.2	14.9
	3' half	13.7	14.6	13.4	15.2	14.3	15.1
	Difference^e^	0	0.3	-0.1	0	0.1	0.2
**YHB1 **(*cleavage at the first and last thirds*)^c^	5' third	14	13.6	11.9	13.7	13.4	14.8
	3' third	13.5	13.2	11.4	14.1	12.6	14.5
	Difference^e^	-0.5	-0.4	-0.5	0.4	-0.8	-0.3
**YGR026W **(*cleavage at the middle of the ORF*)^d^	5' half	12.4	14.7	13.8	13.1	12.9	15.4
	3' half	12.5	14.9	13.9	13.5	12.6	15.4
	Difference^e^	0.1	0.2	0.1	0.4	-0.3	0

**Figure 5:**
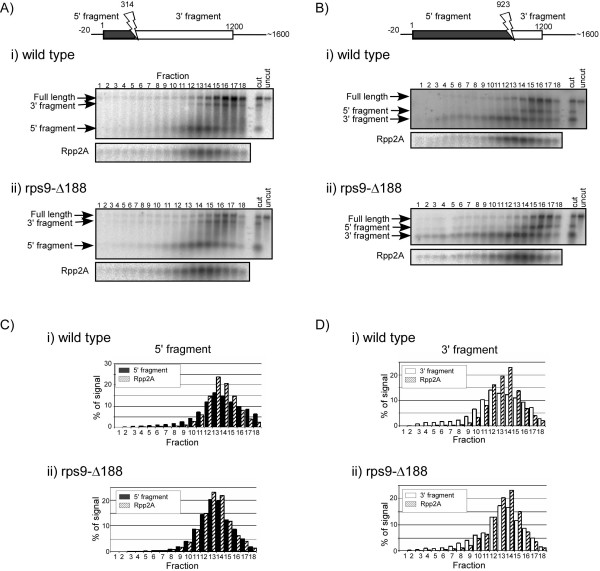
**Comparison of ribosomal association between two ends of an mRNA by RDM**. A and B) Yhb1 mRNA was subjected to RDM analysis by addition of RNase H and ODN complementary to a region 314 nts (A) or 923 nts (B) downstream to the start codon (indicated by a lightning-symbol on the schematic presentation of Yhb1 mRNA). Cleavage by the RNase H and ODN at these positions is expected to yield a fragment containing the 5' third of the ORF (depicted in black) and a fragment containing the 3' third of the ORF (depicted in white). The RDM was performed on a fraction isolated from wild-type (panel i) or *rps9-Δ188 *(panel ii) strains. Unrelated polysomal fraction containing mRNAs associated with three ribosomes was added to each sample at the end of the reaction to serve as a common reference for the following separation step. Following the RNase H cleavage, samples were separated on a sucrose gradient into 18 fractions and subjected to northern blotting. The blots were first hybridized with Yhb1 probe (upper blots in each panel) and then with a probe to Rpp2A mRNA that sediments as associated with three ribosomes (lower blot in each panel). Arrows to the left of each panel indicate the migration position of the cleavage products as well as residual uncut mRNA ("full-length"). C) Quantitation results of the northern blots presented in A. Hatched bars in all panels present the signals of Rpp2A and black bars present the signal of the 5' fragment. D) Quantitation results of the northern blots presented in B. Hatched bars in all panels present the signals of Rpp2A and white bars present the signal of the 3' fragment.

Numbers indicate the weighted-average fraction for each fragment. They were obtained by summing the products of the relative signal in a fraction times the fraction number for all fractions with a signal above background.

a – This experimental repeat is presented in Figures 4 and 5.

b – Cleavage scheme as depicted in Figure 4A.

c – Cleavage scheme as depicted in Figure 5. Note that in the second and third repeats, the cleavage was done simultaneously with both ODNs added to the same sample. This enabled performing the entire procedure with a single sample (including separation on a single sucrose gradient and using northern analysis), therefore lowering the experimental error.

d – Cleavage scheme as depicted in Figure 4B.

e – Difference between the weighted-average fraction for the 3' fragment and the 5' fragment. This value represents the difference in sedimentation between the two fragments.

We analyzed another two mRNAs (*Nop15 *and *Aro7*) for differences in sedimentation between their 3' and 5' halves. The results suggest that on these mRNAs as well there is an increased density of the 3' half in the mutant strain, yet the extent of increase is small and therefore its significance is unclear (Additional File 8).

To further pinpoint the region of increased density, we designed RDM for a shorter region towards the stop codon of *Yhb1 *mRNA. Polysomal RNA was mixed with an ODN complementary to a region 277 nts upstream to the stop codon. For comparison, ODN complementary to a region 314 nts downstream to the start codon was used in a parallel reaction (Fig. 5). These cleavage positions are expected to result in fragments representing the 5' third of the ORF (cleavage at position 314) and the 3' third of the ORF (cleavage at position 923). Each of the reactions (position 314 or 923 in either the wild-type or mutant strains) was separated on a sucrose gradient. Since in this experimental set-up each sample is separated in a different gradient, a fraction of mRNAs associated with three ribosomes was collected, and an aliquot from it was added to each sample to serve as a common reference for the separation step. Following separation, multiple fractions were collected, and the sedimentation of the 3' and 5' products was identified using specific probes (Fig. 5). In addition, the sedimentation position of Rpp2A mRNA, which normally sediments in the fraction of three ribosomes, was determined using a specific probe and served as a reference for all gradients. Quantitation of the sedimentation of the 5' fragments from the wild-type and mutant strains revealed a sedimentation similar to Rpp2A mRNA (Fig. 5A, upper and lower graphs); in both cases, the maximal signal for *Rpp2A *(hatched bars) and the 5' fragments (black bars) appeared in fractions 13–14 (Fig. 5C). However, the 3' fragments appear to sediment differentially compared to *Rpp2A *(Fig. 5B). In the wild-type strain a two fraction difference is observed between the peak fractions of *Rpp2A *(hatched bars) and the 3' cleavage product (white bars) (fractions 14 and 12, respectively), yet in *rps9-Δ188 *only a one fraction difference is noted (fractions 14 for Rpp2A and 13 for the cleavage product (Fig. 5D)). We also performed this experiment in a slightly modified protocol, whereby both ODNs were added to the same cleavage sample and the products were separated on a single sucrose gradient. This set-up allowed direct comparison of their sedimentations without the need for an external reference (i.e., Rpp2) (Table 4). In all cases, the 3' fragment (which includes ~300 nts upstream to the stop codon) sedimented in heavier fractions than a similar size 5' fragment. These results further suggest that there is an increased ribosomal association towards the 3' end of *Yhb1 *mRNA in *rps9-Δ188 *strain.

### *rps9-Δ188 *does not confer a read-through phenotype

**Figure 6:**
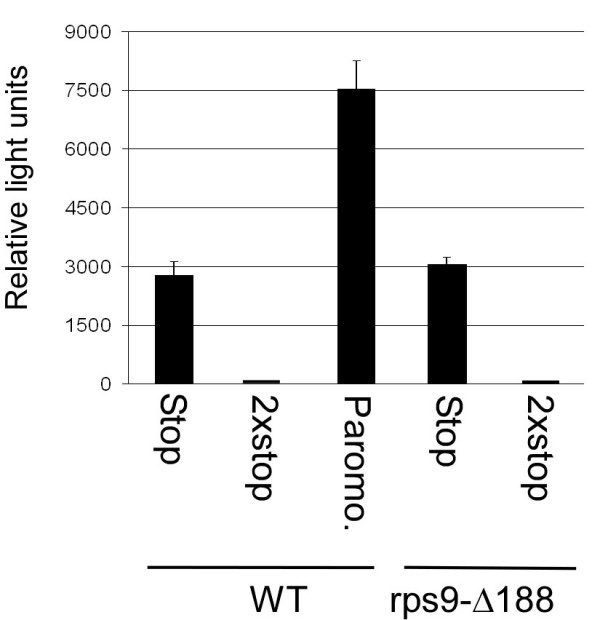
**Luciferase assay to measure read-through efficiency**. Parental strain (WT) or *rps9-Δ188 *strain were transformed with a plasmid carrying the luciferase gene with a single premature stop codon ("Stop") or two consecutive stop codons ("2× stop"). Luciferase activity was measured after the induction of luciferase expression by 2 hr growth in medium containing galactose. A sample of cells carrying the premature stop codon was treated with paromomycin for 90 min to induce read-through efficiency. Note that the activity of a normal luciferase gene under these experimental conditions was at least 100 times higher than the single premature stop gene (data not shown).

A possible basis for increased density at the 3' end is read-through of ribosomes into the 3' UTR. This is a compelling possibility in the case of S9 since some mutations in it were previously shown to yield read-through of premature termination codons (PTC) [3,4,7]. To quantitatively analyze the efficiency of read-through in *rps9-Δ188*, we used a reporter assay in which the activity of a PTC-containing luciferase gene was tested [8]. Plasmids carrying the luciferase gene with one PTC ("stop") or two PTC ("2× stop") under the control of an inducible promoter (*Gal1*) were transformed into either the wild-type or the *rps9-Δ188 *strains. Luciferase activity in these strains was measured after 2 hr induction of expression in galactose. As can be seen in Figure 6, some read-through activity is observed in the wild-type strain. This activity is eliminated completely if there are two consecutive stop codons, and is induced by more than two-fold following treatment with paromomycin. In the *rps9-Δ188 *strain, read-through levels are similar to those in the wild-type, indicating that the S9 mutation does not confer a read-through phenotype to a pre-mature stop codon.

## Discussion

### Genome-wide analysis of the role of ribosomal proteins

Ribosomal proteins are known to play many roles during the translation process [1,2]. Various methods were used to explore these roles, including sensitivity assays to antibiotics, analyses of reporter mRNAs carrying premature termination codons or rare codons, and structural assays that examined their positions on the ribosomes at different translation states. Here we took a different approach and used the information extracted from the ribosomal association of many different sequences in order to gain insight into the role of a particular ribosomal protein. We were able to compare the ribosomal association of ~800 mRNAs and divide them into several groups of affected mRNAs. Our limited analysis did not reveal any physical or functional feature that correlated with the extent of change (Table 3). Yet, our data might serve as a basis for more thorough analyses, utilizing novel computational tools. Most necessary are algorithms for calculating and aligning RNA structures, which may reveal correlations that we failed to identify. Another current limitation stems from the limited information available for various mRNA features, such as 3' UTRs length, local structure stability and sequence elements. Recently, a large-scale cDNA analysis provided important information about lengths of 5'UTRs in yeast [26]. Although we did not find correlation between the 5' UTRs lengths and changes in ribosomal association, information other than the length (e.g., structural elements) may be involved. Better characterization of these features may assist in defining broader roles of S9.

Changes in transcript levels were shown in many cases to correlate with changes at the translational level, a phenomenon that was termed 'potentiation' [27]. Such a process can occur, for example, if specific proteins are deposited on the transcript as it is synthesized [28], or if alternative transcription start site is selected [29]. When comparing the changes in transcriptome to the changes in ribosome association of the 815 genes, we did not observe any correlation between the two (data not shown). This could be because our sample is relatively small and has small changes in relative transcript levels. Alternatively, the effects of S9 might only be at the translation step (e.g., during translation termination), and therefore the changes of transcript levels are only indirect consequences of the translational effects. Such indirect secondary effects are likely to be diverse, therefore simple correlation will not be observed.

The analysis presented here focused on those mRNAs having increased density, and based on the few mRNAs that were tested specifically, we propose a role for S9 in termination. However, a significant number of genes appeared to have a similar ribosomal association as in the wild-type, as well as decreased density compared to the wild-type strain. This suggests that the effects on the termination of S9 are conferred only to a subset of mRNAs, for example, mRNAs with a specific sequence or structural motif in their 3' UTR. We were unable to identify a common sequence motif using standard tools (e.g., MEME, [30]) and are currently attempting to combine structure alignment algorithms to this end. Alternatively, S9 might similarly affect termination on all mRNAs. Yet, due to differences in re-initiation rates between different mRNAs, the effect will appear to be differential and strong enough on some mRNAs to lower ribosomes' reloading. In such a model, the net balance of termination inhibition will be a decrease in ribosomal association for mRNAs with a strong affinity between their 5' and 3' ends, and an increase in density for mRNAs with weak interactions. Testing this model necessitates knowledge about affinities between the ends of different mRNAs or the rates of re-initiation on different mRNAs. Such information is yet to be determined.

### Possible involvement of S9 in termination

We have herein focused on the sub-group of mRNAs having increased ribosomal association. For two of the tested mRNAs an increased ribosomal association towards the 3' end of the mRNA was observed; such an increase cannot be excluded for the other two mRNAs. The increased density towards the 3' end of the mRNA might be due to ribosomes reading through the stop codon and accumulating at the 3' UTR. However, if our results from the premature termination codon analysis (Fig. 6) are applicable to a natural stop codon, a likely explanation for the increased density is that ribosomes accumulate upstream to the stop codon. S9 might interact with one of the elongation or release factors that are necessary for efficient termination and ribosome recycling [31-33].

### Possible roles of the acidic domain

The region of S9 that was changed includes multiple acidic amino acids. The high-resolution structures of both the prokaryotic and the eukaryotic ribosomes suggest that this region can interact with incoming mRNA or other proteins. There are numerous examples of protein-RNA interactions that are performed through negatively charged residues [34]. These residues interact preferentially with the bases of the mRNA molecule [35]. It is possible that once the ribosome approaches the termination region, the C terminus of S9 interacts with the incoming 3' UTR and thereby allows efficient termination. Another possible role for the acidic domain is through interaction with other proteins. The C terminus might interact with elongation or termination factors, or with other proteins necessary for efficient termination.

## Conclusion

We presented here the first genome-wide study of changes in ribosomal association due to a mutation in the ribosomal protein (S9). For analysis we selected a protein that resides at the entrance of the mRNA leading into the ribosomes and which might interact with the incoming message. This analysis revealed groups of mRNAs that changed their ribosomal association compared to the wild-type strain. Limited bioinformatics analysis did not reveal a general correlation between the change in association and physical or functional properties of the mRNA (Table 3). Yet, this data might serve as a template for the development of more advanced tools that will identify a novel sequence or structure elements in the mRNA. We focused here on the group of mRNAs having increased association and found that at least for some the increased association is biased towards the 3' end of the transcript. This suggested a novel function for S9 in the termination process. Future studies aimed at identifying elements or factors that interact with S9 will provide insight into the exact mechanism by which S9 affects termination.

## Methods

### Strains and growth conditions

Strains are listed in Table 1. To allow construction of *rps9a *and *rps9b *double knockout (strain YA178), haploid strains deleted of either 9A (Euroscarf Y02747) or 9B (Euroscarf Y16961) were mated (to allow selection for deletion of 9B, the kanMX4 in strain Y16961 was replaced by HIS3). The diploid was transformed with a plasmid expressing *rps9-Δ188 *from its native promoter and with its 3' UTR. This strain was then sporulated and cells that were deleted of rpS9A, 9B and expressed *rps9-Δ188 *were selected on the appropriate medium.

**Table 1:** List of strains

**Strain**	**Relevant Genotype**	**Reference**
BY4741	*MAT *a *his3Δ1 leu2Δ0 met15Δ0 ura3Δ0*	Euroscarf
Y02747	BY4741 *MAT *a *his3Δ1 leu2Δ0 met15Δ0 ura3Δ0 rps9a::kanMX4*	Euroscarf
Y16961	BY4742 *MAT *á *his3Δ1 leu2Δ0 lys2Δ0 ura3Δ0 rps9b::kanMX4*	Euroscarf
YA178	BY4741 *MAT *a *his3Δ1 leu2Δ0 met15Δ0 ura3Δ0 rps9a::kanMX4::HIS3 rps9b::kanMX4 shm2::kanMX4::hisG +PA183(rps9-Δ188) +PA185*	This study
YA223	*MAT *a *his3Δ1 leu2Δ0 met15Δ0 ura3Δ0 *+PA27 (*luciferase-stop*)	This study
YA226	BY4741 *MAT *a *his3Δ1 leu2Δ0 met15Δ0 ura3Δ0 rps9a::kanMX4::HIS3 rps9b::kanMX4 shm2::kanMX4::hisG +PA183(rps9-Δ188*)+ *PA27 (luciferase-stop*)	This study
YA227	BY4741 *MAT *a *his3Δ1 leu2Δ0 met15Δ0 ura3Δ0 rps9a::kanMX4::HIS3 rps9b::kanMX4 shm2::kanMX4::hisG +PA183(rps9-Δ188) *+PA28 (*luciferase 2 × stop*)	This study

The *rps9-Δ188 *strain also carried a deletion of the *Shm2 *gene and expressed a Flag-tagged *Shm2 *controlled by its own promoter from a plasmid. This modification was included to allow future studies in which modification of a reporter mRNA will be necessary. *Shm2 *is a highly expressed, non-essential gene that is involved in the one-carbon metabolism. It does not have any known role in translation or ribosome function.

Mating, sporulation and lithium acetate transformation were all carried out using conventional methods [36]. Yeast was grown normally to the mid-logarithmic phase either in rich media (YPD) or selective media (SD with the relevant drop outs).

### Plasmids

The plasmids used are listed in Table 2. *RPS9 *constructs were made by PCR amplification of a genomic region starting from 500 bp upstream to the *RPS9A *gene and ending 300 bp downstream to the stop codon. This yielded a PCR product that is 1,895 bp long and includes the entire *RPS9A *ORF with its intron, 500 bp upstream and 300 bp downstream. This fragment was inserted into pRS415 and pRS416 vectors, and served as a template for the construction of *rps9-Δ188*. *rps9-Δ188 *was constructed by Hind3 cleavage, Klenow filling-in and religation of the plasmid. All constructs were confirmed by sequencing.

**Table 2:** List of plasmids

**Plasmid**	**Vector**	**Insert**
PA27	pRS216	*pGal1-luciferase lys445TAG*
PA28	pRS216	*pGal1-luciferase lys445TAG TAA*
PA111	pRS415	*Flag-S9*
PA170	pRS415	*Flag-rps9-Δ188*
PA183	pRS415	*rps9-Δ188*
PA184	pRS416	*rps9-Δ188*
PA185	pRS416	*Flag-Shm2*

### Western analysis

Protein extracts were prepared from trichloroacetic acid-treated yeast cells as previously described [37]. Immunoblot procedures were essentially as described [38] using mouse monoclonal antibodies directed against Flag (Sigma cat. no. F3165, 1:5000 dilution).

### Luciferase assay

Luciferase plasmids were kindly provided by Dr. Daniel Reines [8] and inserted into the relevant strains (Table 1). Cells were grown to the mid-logarithmic phase, and the expression of luciferase genes was induced by 2 hr growth in galactose. Paromomycin treatment was done by the addition of paromomycin (200 μM final concentration) 30 min after the shift to galacose, and cells were grown for an additional 90 min. Luciferase assay was performed using the Luciferase Assay System from Promega (Cat. E1500).

### RNA methods

Yeast total RNA was extracted by the hot phenol extraction method. Extraction procedure for polysomal preparation was essentially as described [39] except that the lysis was performed by two rounds of 1.5 min treatment in a bead beater. RDM analysis was performed as described [40]. Briefly, 50 ml of BY4741 cells were grown to OD_600 _0.6–0.9 at 30°C in YPD medium (1% yeast extract, 2% Bacto peptone, 2% glucose). Cells were harvested following cycloheximide (CHX) addition to the medium to a final concentration of 0.1 mg/ml, and lysed as previously described [39]. Lysate was separated through a sucrose gradient and the fractions containing the majority of the tested mRNA were collected. Standard fraction volume was ~600 μl, into which DTT and Ribonuclease Inhibitor (Porcine liver, Takara) were added to a final concentration of 0.15 mM and 400 units/ml, respectively. Oligodeoxynucleotides (ODN) complementary to the regions of interest were added to a final concentration of 0.16 μM and annealing was allowed for 20 min with a gradual decrease in temperature from 37°C to room temperature. Cleavage reactions were initiated by the addition of RNase H (10 units per reaction) and 5× RNase H buffer (0.1 M Tris pH 7.4, 0.5 M KCl, 0.1 M MgCl_2_, 0.5 mM DTT, 2.5 mg/ml CHX). Reactions were allowed for 30 min at 37°C and terminated by the addition of 400 μl of LMD (20 mM Tris pH 7.4, 140 mM KCl, 1.5 mM MgCl_2_, 0.5 mM DTT, 0.1 mg/ml CHX and 1 mg/ml Heparin). Cleavage products were then separated by velocity sedimentation on a 10–50% sucrose gradient, and fractions were collected into tubes containing 1.5 fraction volumes of 8 M Guanidium HCl and 2.5 fraction volume of 100% ethanol. Pallets were resuspended and the sedimentation position of each cleavage product was determined by northern analysis [41]. Radioactive probes were prepared by random priming incorporation of ^32^P-labled nucleotides into a PCR fragment homologous to the tested mRNA.

### Microarrays production and analysis

DNA microarrays were produced using a contact spotter (built according to the MGuide, Stanford). All known and predicted yeast ORFs (~6,000) were PCR-amplified from start to stop codon using specific primers. Samples of each reaction were analyzed by gel electrophoresis and the remainder was precipitated and used for spotting on Amino silane coated slides (GAPS II from Corning). Following hybridization, microarrays were scanned by an Axon 4000B scanner, and spots were analyzed using the GenePix 5.1 program (Axon Instruments). Low-quality spots were filtered out if they were not distinguishable from the local background (difference less than 300), or had large variations in pixels' ratio (correlation coefficient less than 0.5). Polysomal analysis normalization was done by the addition of equal amounts of five different *in vitro *transcribed *B. subtilis *RNAs to each of the fractions immediately after collection [19]. DNA complementary to these RNAs was spotted at least 50 times on the DNA microarray, and the signals obtained from these spots were used to normalize any global differences in the signals of different microarrays [20]. For transcriptome analysis, normalization was done according to the median ratio of all spots on the microarray.

### Peak fraction and ribosomal association assignment

Three independent experimental repeats carrying out the entire protocol from yeast growth through polysomal fractions and microarray hybridization were performed. The fraction collection parameters led to 13 or 14 fractions per collection (variation due to small differences in gradient volume). In each repeat, all of the fractions corresponding to complexes smaller than 80S (i.e., mRNA free of ribosomes) were combined and hybridized together to the DNA microarray. The rest of the fractions were not combined and were hybridized individually to DNA microarrays. Every experiment therefore includes 8 to 10 microarrays, each representing a different region of the gradient. Following normalization of the DNA microarrays signals, red-to-green ratios were obtained for each gene from the different microarrays to generate its distribution profile along the gradient. In many cases, the distribution profile included missing values, namely a signal that did not pass the quality selection in a specific microarray. For further analysis, we selected only those genes that had only a single missing value, unless it appeared in one of the extreme fractions, in which case it was not counted as missing but assumed to contain very low amounts of mRNA (Additional File 3). An exception to this single missing value rule was done in the first repeat, where fraction number 10 gave a very poor signal and the entire fraction was excluded from the analysis. This fraction included mRNAs associated with two ribosomes, and analysis of the two other repeats revealed that almost none of the mRNAs exhibited their highest association with two ribosomes.

To determine the number of ribosomes that a transcript is mostly associated with, we first defined the peak fraction of sedimentation, which is the polysomal fraction where the highest number of each gene's mRNAs sediments. In each repeat for each gene, the fraction (excluding the free fraction) with the highest ratio was defined as the peak fraction. The number of ribosomes sedimenting in the peak fraction was deduced directly from peaks in the OD_254 _profile of the gradient. For fractions derived from the limited resolution region of the gradient (fractions 13 and 14), the number of ribosomes was extrapolated from the high-resolution region (see Additional File 2). Next, the number of ribosomes obtained from the three repeats was averaged to yield the average number of ribosomes on a transcript (Additional File 4). Averaging was done only if two or more experimental repeats appeared to deviate by one or less fractions. Cases where the peak fraction appeared to be a single ribosome were not included in the averaging unless they were either one or two ribosomes in all three repeats. The exclusion of repeats in which the peak appeared in the single ribosome fraction was based on the assumption that mRNAs with a single ribosome represented a different translational status than mRNAs in polysomes.

## Authors' contributions

LP performed the experiments and analyzed the data. YA performed some of the data analysis and wrote the manuscript. All authors read and approved the final manuscript.

## Supplementary Material

Additional file 1 Microarray results of the transcriptome analysis. Excel file with the results of the transcriptome analysis of wild type and mutant strains grown either in YPD (Rich media) or SD (minimal media). Click here for file

Additional file 2 All microarray results of polysomal fractions. Excel file with the results of the microarray analysis of all polysomal fractions, from the three experimental repeats. Click here for file

Additional file 3 Genes that passed filtration criteria. Excel file that contains the list of genes that passed the "missing value" selection. Click here for file

Additional file 4 Average number of ribosomes per gene. Excel file that contains a list of genes with their average number of ribosomes (after exclusion of genes with large variations between repeats). Click here for file

Additional file 5 Number of ribosomes per gene in the wild type and mutant strains. Excel file with the list of genes that had a corresponding ribosomal association in the wild type strain. This file also includes various mRNA features for each gene. Click here for file

Additional file 6 GO term analysis for genes with a reduced number of ribosomes. Excel file with the results of the GO term analysis for the genes that experienced a decrease of two or more ribosomes in the mutant strain. Click here for file

Additional file 7 GO term analysis for genes with an increased number of ribosomes. Excel file with the results of the GO term analysis for the genes that experienced an increase of two or more ribosomes in the mutant strain. Click here for file

Additional file 8 Sedimentation pattern of the 5' and 3' halves of NOP15 and ARO7 in *rps9-Δ188 *cells and wild type cells. RDM results for two additional mRNAs that appeared to have an increase in ribosomal association in the mutant strain. Click here for file
