# Supplementary figures and images for: Genome-wide polysomal analysis of a yeast strain with mutated ribosomal protein S9
Source: BMC Genomics. 2007 Aug 21;8:285. doi: 10.1186/1471-2164-8-285 (PMC2020489; doi:10.1186/1471-2164-8-285)

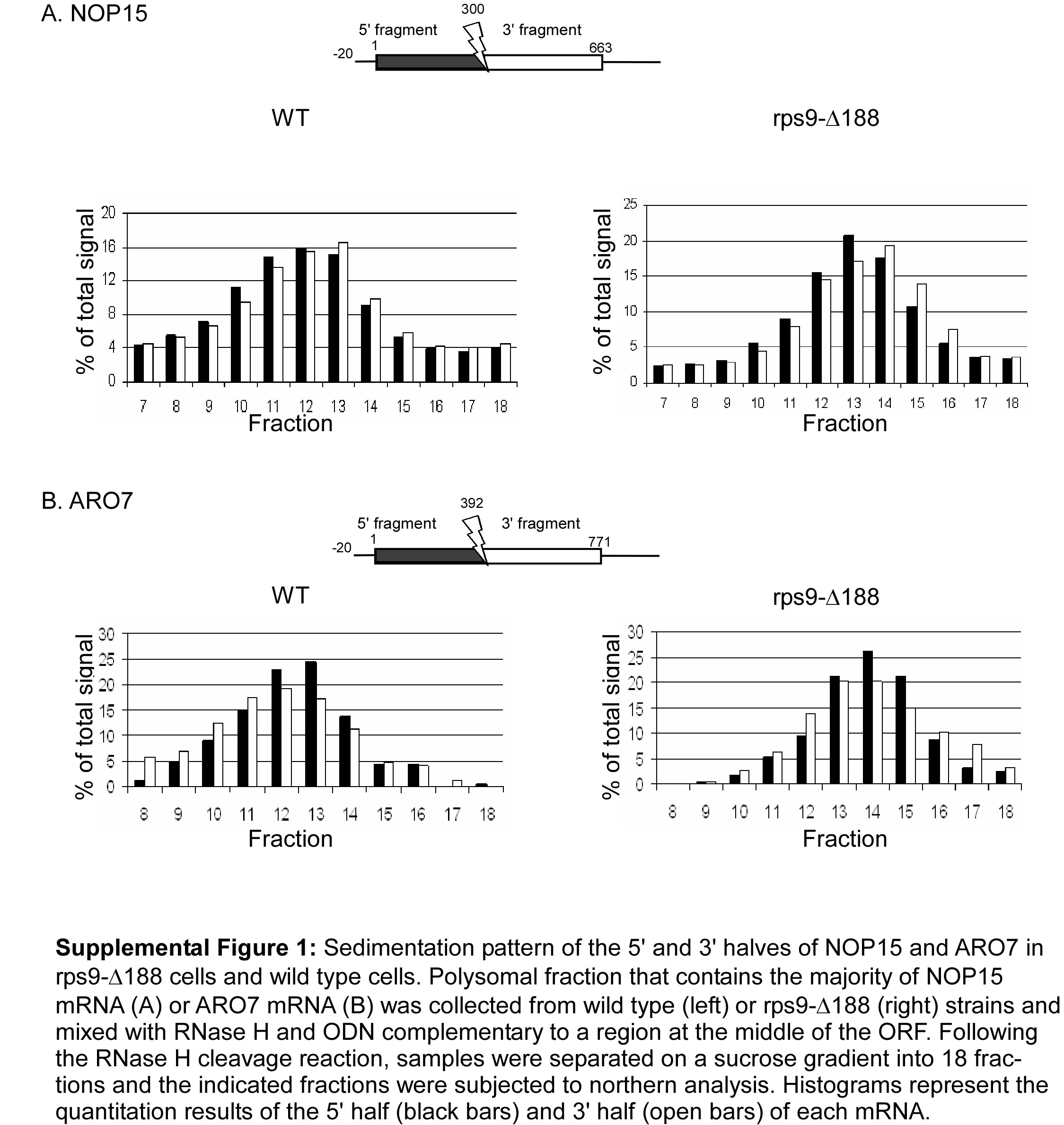

Supplement: Additional file 8 — Sedimentation pattern of the 5' and 3' halves of NOP15 and ARO7 in rps9-Δ188 cells and wild type cells. RDM results for two additional mRNAs that appeared to have an increase in ribosomal association in the mutant strain. [file 1471-2164-8-285-S8.jpeg]
